# Trunk Rotation Isokinetic Strength Changes During a 15-Week Periodized Strength Training Program in Three Chinese Elite Paralympic and Deaflympic Throwers

**DOI:** 10.3390/jfmk11040389

**Published:** 2026-09-28

**Authors:** Muzu Hou, Haifeng Ma

**Affiliations:** 1 School of Competitive Sports, Shanghai University of Sport, Shanghai 200438, China; hmzel24@sus.edu.cn; 2 Key Laboratory of Sports Performance Evaluation and Monitoring, Shanghai University of Sport, Shanghai 200438, China

**Keywords:** Paralympic and Deaflympic throwers, trunk rotation, isokinetic strength, periodized training, Cohen’s d, case series

## Abstract

**Background**: Trunk rotation strength is a key performance factor in throwing events, but periodized training effects in elite Paralympic and Deaflympic throwers are sparsely documented. **Methods**: Three Chinese elite throwers (one F35 cerebral palsy, one F60 hearing impairment, and one F11 visual impairment) completed a 15-week Bompa-based periodized strength program (3 November 2018 to 16 February 2019). Trunk rotation peak torque (PT) and peak power (PP) were measured on a Biodex System 4 dynamometer at four angular velocities (60, 120, 150, and 180°/s) before and after the intervention. **Results**: Effect sizes for trunk rotation peak torque and peak power ranged from negligible to large (d = 0.05–2.93). The F35 athlete showed a decline in left-rotation peak torque at 180°/s (270.73 → 260.40 N·m, d = 0.64) and persistent left-right asymmetry. The F60 and F11 athletes showed predominantly positive adaptations across velocities. Due to the small heterogeneous sample (*n* = 3) and absence of a control group, these findings are hypothesis-generating rather than confirmatory. Throwing performance was not measured. **Conclusions**: The program is descriptively feasible across F35/F60/F11 classifications, but findings are hypothesis-generating given the absence of a control group and the small heterogeneous sample. Throwing performance was not measured.

## 1. Introduction

Trunk rotation muscle strength is a recognized performance factor in throwing events [1]. Studies on able-bodied athletes—tennis players [2], golfers [3], and throwers [4]—have shown that trunk rotation strength correlates with throwing performance, with some differences between the dominant and non-dominant sides [5,6,7,8,9]. For athletes with motor, sensory, or visual impairments, the relationship between trunk rotation strength and throwing performance is less clear, and the few existing studies are predominantly single-case or small-sample reports [5,6,7,8,9].

Bompa’s periodization framework [10,11,12,13], which structures training across macro-, meso-, and micro-cycles, has been adapted for Paralympic and Deaflympic sport [14], but its empirical validation in these populations is limited. The available evidence base is therefore not amenable to group-level inferential synthesis, and a descriptive case-series design is the appropriate methodological standard.

We evaluated the effects of a 15-week periodized strength training program on trunk rotation isokinetic strength in three elite Chinese Paralympic and Deaflympic throwers, with three aims: (1) describe individual changes in peak torque and peak power [15] at four angular velocities; (2) describe left-right rotational asymmetry before and after the intervention; (3) explore whether sport-class-specific patterns emerge that could inform future controlled studies.

## 2. Methods

Three Chinese elite throwers (one male, two female) participated during the winter preparation period (3 November 2018 to 16 February 2019). Athlete 1 (Fu, male, 35 yr, F35 cerebral palsy) competes in Paralympic shot put and discus. Athlete 2 (Ren, female, 27 yr, F60 conductive hearing loss) competes in Deaflympics shot put. Athlete 3 (Zhang, female, 26 yr, F11 visual impairment) competes in Paralympic shot put and javelin. The combination of age, sex, sport class, event, and gold-medal history makes the athletes readily identifiable despite the use of surnames only; written informed consent specifically covered publication of this potentially identifiable information. The large population of elite Paralympic and Deaflympic throwers is small (fewer than 50 per classification worldwide) [16], and the heterogeneity of impairment profiles precludes aggregation; multi-case study designs are the appropriate standard in this context [17,18].

### 2.1. Training Periodization

The 15-week program was structured into general preparation (weeks 1–3, 25 sessions, 60–70% general/30–40% specific) and specific preparation (weeks 4–15, 104 sessions, 30–40% general/60–70% specific). The 129 total sessions comprised strength training (45, 3×/week Mon/Wed/Fri), throwing (30), conditioning (30), and other training (24). Strength training followed three sequential mesocycles: MC1 anatomical adaptation (weeks 4–6), MC2 maximal strength (weeks 7–11), MC3 sport-specific strength conversion (weeks 12–15). The schedule for the winter training period is in Table 1,and athlete-specific modifications are in Table 2. Detailed schedule about the training plan is shown in Supplementary Tables S1 and S2.

### 2.2. Testing Protocol

Trunk rotation isokinetic strength was assessed pre-intervention (2 November 2018) and post-intervention (19 February 2019) on a Biodex System 4 dynamometer (Biodex Medical Systems, Shirley, NY, USA) [19,20]. After a 5-min warm-up and 7-day familiarization period (3 submaximal + 2 maximal contractions at each velocity), each athlete performed 3–5 maximal concentric trunk rotation contractions at 60, 120, 150, and 180°/s in randomized order, with 60-s rest between velocities. Range of motion was ±45° from anatomical neutral. The single highest peak torque (N·m) and peak power (W) at each velocity and direction were retained for analysis [21,22]. Calibration was performed before each test session per manufacturer protocol [23].

### 2.3. Statistical Analysis

We report individual descriptive data: pre and post mean ± SD, absolute change (Δ), percentage change (%Δ), Cohen’s d effect size with magnitude thresholds (negligible |d| < 0.2, small 0.2–0.5, medium 0.5–0.8, large ≥ 0.8) [24], and 95% CI for d calculated as d ± 1.96 × SE_d, where SE_d = sqrt(1/*n* + d^2^/(2*n*)) with *n* = 3. No inferential tests were conducted. Confidence intervals at *n* = 3 are unstable and should be interpreted with caution. Per-repetition peak values were used (rather than the mean of multiple repetitions), a design choice that introduces a pseudo-replication concern (see Section 4.3). Analyses were conducted in IBM SPSS Statistics 25.0 and Microsoft Excel 2016.

## 3. Results

### 3.1. Peak Torque

The peak torque of trunk rotation before and after the 15-week periodized strength training program is shown in Table 3.

Athlete 1 (Fu, F35). At 60°/s, left PT increased from 232.07 ± 10.30 to 240.33 ± 9.96 N·m (Δ = +8.27, %Δ = +3.56%, d = 0.80) and right PT from 170.27 ± 6.55 to 174.50 ± 5.91 N·m (Δ = +4.23, %Δ = +2.49%, d = 0.64). At 120°/s, left PT increased from 256.67 ± 21.30 to 267.50 ± 19.20 N·m (Δ = +10.83, %Δ = +4.22%, d = 0.51) and right PT from 184.83 ± 15.10 to 193.20 ± 14.10 N·m (Δ = +8.37, %Δ = +4.53%, d = 0.56). At 150°/s, left PT was essentially unchanged (274.47 → 271.07 N·m, %Δ = −1.24%, d = 0.16) while right PT increased from 187.17 ± 14.20 to 198.13 ± 13.50 N·m (%Δ = +5.85%, d = 0.77). At 180°/s, left PT decreased from 270.73 ± 8.30 to 260.40 ± 7.50 N·m (%Δ = −3.82%, d = 0.64) while right PT increased from 199.07 ± 10.20 to 206.30 ± 9.80 N·m (%Δ = +3.63%, d = 0.72). Fu’s high-velocity left-rotation PT reduction is the most notable individual finding in this case.

Athlete 2 (Ren, F60). At 60°/s, left PT increased from 167.93 ± 14.36 to 179.33 ± 11.98 N·m (%Δ = +6.79%, d = 0.79) and right PT from 130.53 ± 6.20 to 133.57 ± 5.80 N·m (%Δ = +2.33%, d = 0.49). At 120°/s, left PT increased from 184.20 ± 18.50 to 188.80 ± 16.40 N·m (%Δ = +2.50%, d = 0.25) and right PT from 145.57 ± 7.80 to 152.40 ± 7.10 N·m (%Δ = +4.69%, d = 0.88). At 150°/s, left PT increased from 174.80 ± 12.20 to 189.67 ± 11.10 N·m (%Δ = +8.51%, d = 1.22) and right PT from 145.93 ± 4.50 to 154.87 ± 4.30 N·m (%Δ = +6.12%, d = 1.99). At 180°/s, left PT increased from 168.77 ± 14.10 to 177.50 ± 12.80 N·m (%Δ = +5.17%, d = 0.62) and right PT from 141.43 ± 10.30 to 146.80 ± 9.50 N·m (%Δ = +3.80%, d = 0.52). Ren’s peak PT occurred at 120°/s, which is the lowest velocity of the three athletes.

Athlete 3 (Zhang, F11). At 60°/s, left PT increased from 171.50 ± 13.10 to 180.38 ± 12.20 N·m (%Δ = +5.18%, d = 0.67) and right PT from 152.88 ± 13.70 to 166.63 ± 12.50 N·m (%Δ = +9.01%, d = 0.99). At 120°/s, left PT increased from 180.30 ± 15.40 to 185.50 ± 14.20 N·m (%Δ = +2.88%, d = 0.34) and right PT from 168.77 ± 12.60 to 172.30 ± 11.80 N·m (%Δ = +2.09%, d = 0.28). At 150°/s, left PT increased from 174.50 ± 16.30 to 184.90 ± 15.10 N·m (%Δ = +5.96%, d = 0.64) and right PT from 168.93 ± 13.40 to 176.30 ± 12.50 N·m (%Δ = +4.36%, d = 0.56). At 180°/s, left PT was essentially unchanged (172.83 → 173.80 N·m, %Δ = +0.56%, d = 0.11) and right PT increased from 167.77 ± 13.20 to 175.13 ± 12.20 N·m (%Δ = +4.38%, d = 0.56). Zhang’s data were the most uniform across the velocity spectrum, with no negative individual changes.

### 3.2. Peak Power

The peak power of trunk rotation before and after the 15-week periodized strength training program is shown in Table 4.

Athlete 1 (Fu). At 60°/s, both sides showed large positive changes (left: 127.13 → 148.27 W, %Δ = +16.63%, d = 2.38; right: 79.73 → 100.13 W, %Δ = +25.59%, d = 2.93). At 120°/s, left increased (140.10 → 156.60 W, %Δ = +11.78%, d = 0.69) and right increased (89.30 → 122.30 W, %Δ = +36.96%, d = 1.83). At 150°/s, left was essentially unchanged (164.07 → 165.10 W, %Δ = +0.63%, d = 0.05) and right increased (118.23 → 137.50 W, %Δ = +16.28%, d = 1.16). At 180°/s, left increased (158.17 → 164.40 W, %Δ = +3.94%, d = 0.38) and right increased (114.40 → 137.07 W, %Δ = +19.81%, d = 1.50).

Athlete 2 (Ren). Effect sizes for Ren’s eight peak power comparisons spanned 0.67 to 2.77, with the most pronounced adaptations at 150°/s (left: 79.30 → 105.17 W, d = 2.77; right: 73.60 → 96.93 W, d = 2.13) and 180°/s left (75.10 → 100.17 W, d = 2.68).

Athlete 3 (Zhang). Zhang’s pattern was the most consistent of the three athletes, with effect sizes of 1.06 to 1.86 and the largest adaptations at 150°/s (left: 91.20 → 125.93 W, d = 1.86; right: 79.50 → 110.07 W, d = 1.49).

### 3.3. Left-Right Trunk Rotation Ratios

The trunk rotation ratios between the two sides before and after the 15-week periodized strength training program are shown in Table 5.

All three athletes exhibited higher left-rotation than right-rotation peak torque at all angular velocities, both pre- and post-intervention. Fu showed the most pronounced asymmetry at 180°/s (1.63 post-intervention vs able-bodied reference 1.34 [4]); Ren and Zhang showed smaller but still elevated L/R ratios. These ratios should be interpreted as population-level descriptive indicators, not as clinical or performance thresholds for Paralympic and Deaflympic athletes.

### 3.4. Left-Right Peak Power Differences

Fu’s left/right power differences at 150°/s and 180°/s were 43% and 47% pre-intervention, and 35% and 38% post-intervention, indicating a partial narrowing of the asymmetry. Ren and Zhang showed smaller left/right power differences (5–15%) across all velocities, with no consistent direction of change.

## 4. Discussion

We found descriptive, individual positive changes in trunk rotation PT and PP at most angular velocities in three elite Paralympic and Deaflympic throwers following a 15-week Bompa-based periodized strength program. Effect sizes ranged from negligible to large. Two individual findings warrant attention: the F35 athlete’s high-velocity left PT reduction and the persistent L/R asymmetry despite training.

### 4.1. Comparison with Prior Literature

Zinke et al. [25] reported descriptive improvements in trunk muscle PT and PP in world-class canoe sprinters following a 12-week isokinetic training program, but their study used able-bodied athletes with group-level inferential statistics. Mueller et al. [26] reported improvements in elite adolescent athletes using a randomized controlled pilot trial. Puce et al. [17] reviewed Paralympic powerlifting and identified periodization as a research gap, with most studies using descriptive or single-case designs [17,27,28]. Mechanistic explanations from able-bodied training studies should not be uncritically applied to Paralympic and Deaflympic populations [29,30,31].

### 4.2. Sport-Class-Specific Considerations

The three athletes represented distinct sport classes (F35, F60, F11) with different impairment profiles. Fu (F35, cerebral palsy) showed persistent L/R asymmetry at 180°/s, suggesting that additional bilateral symmetry training may be considered for F35-class throwers. Ren (F60) reached peak PT at 120°/s, lower than the other athletes, suggesting the value of rapid strength and rate-of-force-development work for hearing-impaired throwers. Zhang (F11) showed consistent positive changes across velocities, suggesting the program is feasible for visual-impairment classifications without major modification. These tentative patterns should be confirmed in additional cases.

### 4.3. Limitations

Five limitations should be noted. (1) Small sample (*n* = 3) and absence of a control group preclude attribution of changes to the training program. (2) Single pre-intervention baseline (no multiple baselines) limits separation of training effects from measurement variability. (3) Use of single-repetition peak torque (rather than mean across multiple contractions) introduces pseudo-replication. (4) Heterogeneity of sport classifications (F35, F60, and F11) prevents aggregation. (5) Confidence intervals at *n* = 3 are unstable.

## 5. Practical Applications

Based on the present case-series, three tentative recommendations may be considered: (a) the 15-week three-phase periodized program is descriptively feasible across F35, F60, and F11 sport classifications and may serve as a candidate framework for elite throwers; (b) trunk rotation isokinetic testing at multiple velocities is a candidate component of routine monitoring, useful for characterizing individual velocity-torque profiles; (c) sport-class-specific modifications—including bilateral symmetry work for F35 athletes and rate-of-force-development work for F60 athletes—should be considered, with confirmation in additional cases before adoption.

## 6. Conclusions

A 15-week Bompa-based periodized strength program produced predominantly positive adaptations in trunk rotation peak torque and peak power among three Chinese elite Paralympic and Deaflympic throwers (F35, F60, and F11). The F35 athlete was the exception, showing a decline in left-rotation peak torque at 180°/s and persistent left-right asymmetry. In contrast, the F60 and F11 athletes exhibited consistent gains across most velocities. Given the small, heterogeneous sample and absence of a control group, these findings are hypothesis-generating rather than confirmatory. Future controlled studies—including multiple-baseline or crossover designs with repeated pre-intervention measurements—are needed to test these observations.

## Figures and Tables

**Table 1:** 15-week winter training period.

Period	Weeks	Training Emphasis
General preparation	1–3	Aerobic base, general strength, movement patterning
Specific preparation MC1	4–6	Anatomical adaptation, hypertrophy
Specific preparation MC2	7–11	Maximal strength, power development
Specific preparation MC3	12–15	Sport-specific strength conversion, peak power
Testing	Pre: 2 November; Post: 19 February	Biodex isokinetic trunk rotation testing
Strength sessions	45 total	3×/week (Monday/Wednesday/Friday)
Combined training load	129 total sessions	Strength 45 + Throwing 30 + Conditioning 30 + Other 24

**Table 2:** Detailed training prescription across the three mesocycles.

MC	Weeks	Goal	Exercises	Sets × Reps	Intensity	Rest	Modifications
MC1	4–6	Anatomical adaptation	Back squat	3 × 10	60–65% 1 RM	90 s	
MC1	4–6	Hypertrophy	Romanian deadlift	3 × 10	60% 1 RM	90 s	
MC1	4–6	Trunk rotation	Cable woodchop	3 × 12/side	RPE 6	60 s	Fu: bilateral
MC1	4–6	Throwing pattern	Med ball rotation throw	3 × 8/side	4–6 kg	90 s	
MC1	4–6	Power endurance	Push press	3 × 8	50% 1 RM	90 s	
MC2	7–11	Maximal strength	Back squat	4 × 4	85–90% 1 RM	3 min	
MC2	7–11	Posterior chain	Romanian deadlift	4 × 5	80% 1 RM	3 min	
MC2	7–11	Trunk rotation	Isokinetic trunk rot	4 × 6/side	Max effort	60 s	60–120°/s only
MC2	7–11	Heavy throwing	Shot put from glide	5 × 3	80–85% effort	3 min	Full technique
MC2	7–11	Power	Hang clean	4 × 3	70% 1 RM	3 min	
MC2	7–11	Rate of force	Jump squat	4 × 4	30% 1 RM	3 min	Zhang: emphasis
MC3	12–15	Sport-specific strength	Trap-bar deadlift	4 × 3	85% 1 RM	3 min	
MC3	12–15	Trunk rotation power	Med ball side throw	4 × 5/side	6–8 kg	90 s	Fu: bilateral
MC3	12–15	Velocity-specific	Isokinetic trunk rot	4 × 5/side	Max effort	60 s	All velocities
MC3	12–15	Competition sim	Full throw from circle	5 × 3	90–95% effort	4 min	Ren: visual cue
MC3	12–15	Power peaking	Hang snatch	4 × 3	75% 1 RM	3 min	
MC3	12–15	Rotational stiffness	Pallof press	3 × 8/side	RPE 7	60 s	All athletes
MC3	15	Taper/pre-competition	Back squat	2 × 3	70% 1 RM	3 min	Volume ↓ 50% (50% Volume)
MC3	15	Maintenance	Trunk rotation	2 × 5/side	70% max	60 s	Maintain velocity
MC3	15	Technical refinement	Shot put from glide	4 × 2	95% effort	4 min	Quality focus
MC3	15	CNS activation	Hang clean	2 × 2	60% 1 RM	3 min	Light, fast
MC3	15	Pre-competition prep	Plyometric circuit	2 × 4	Bodyweight	60 s	Activation only

Note: MC1 = Mesocycle 1 (Anatomical Adaptation); MC2 = Mesocycle 2 (Maximal Strength); MC3 = Mesocycle 3 (Sport-Specific Strength Conversion). RPE = Rating of Perceived Exertion (0–10 scale). 1 RM = one-repetition maximum. Athlete-specific modifications address impairment-related considerations (Fu: bilateral emphasis for spastic cerebral palsy; Ren: visual cueing for conductive hearing loss; Zhang: rate-of-force-development emphasis for visual impairment). The downward arrow symbol (↓) herein denotes a decrease in training load.

**Table 3:** Trunk rotation peak torque before and after the 15-week periodized strength training program.

Athlete	Velocity	Direction	Pre (M ± SD)	Post (M ± SD)	%Δ	Cohen’s d	95% CI for d
Fu	60°/s	Left	232.07 ± 10.30	240.33 ± 9.96	+3.56%	0.80 (large)	[−0.50, 2.10]
Fu	60°/s	Right	170.27 ± 6.55	174.50 ± 5.91	+2.49%	0.64 (medium)	[−0.60, 1.88]
Fu	120°/s	Left	256.67 ± 21.30	267.50 ± 19.20	+4.22%	0.51 (medium)	[−0.69, 1.71]
Fu	120°/s	Right	184.83 ± 15.10	193.20 ± 14.10	+4.53%	0.56 (medium)	[−0.66, 1.78]
Fu	150°/s	Left	274.47 ± 17.20	271.07 ± 16.50	−1.24%	0.16 (negligible)	[−0.98, 1.30]
Fu	150°/s	Right	187.17 ± 14.20	198.13 ± 13.50	+5.85%	0.77 (medium)	[−0.52, 2.06]
Fu	180°/s	Left	270.73 ± 8.30	260.40 ± 7.50	−3.82%	0.64 (medium)	[−0.60, 1.88]
Fu	180°/s	Right	199.07 ± 10.20	206.30 ± 9.80	+3.63%	0.72 (medium)	[−0.55, 1.99]
Ren	60°/s	Left	167.93 ± 14.36	179.33 ± 11.98	+6.79%	0.79 (medium)	[−0.51, 2.09]
Ren	60°/s	Right	130.53 ± 6.20	133.57 ± 5.80	+2.33%	0.49 (small)	[−0.71, 1.69]
Ren	120°/s	Left	184.20 ± 18.50	188.80 ± 16.40	+2.50%	0.25 (small)	[−0.90, 1.40]
Ren	120°/s	Right	145.57 ± 7.80	152.40 ± 7.10	+4.69%	0.88 (large)	[−0.45, 2.21]
Ren	150°/s	Left	174.80 ± 12.20	189.67 ± 11.10	+8.51%	1.22 (large)	[−0.27, 2.71]
Ren	150°/s	Right	145.93 ± 4.50	154.87 ± 4.30	+6.12%	1.99 (large)	[0.04, 3.94]
Ren	180°/s	Left	168.77 ± 14.10	177.50 ± 12.80	+5.17%	0.62 (medium)	[−0.62, 1.86]
Ren	180°/s	Right	141.43 ± 10.30	146.80 ± 9.50	+3.80%	0.52 (medium)	[−0.69, 1.73]
Zhang	60°/s	Left	171.50 ± 13.10	180.38 ± 12.20	+5.18%	0.67 (medium)	[−0.58, 1.92]
Zhang	60°/s	Right	152.88 ± 13.70	166.63 ± 12.50	+9.01%	0.99 (large)	[−0.39, 2.37]
Zhang	120°/s	Left	180.30 ± 15.40	185.50 ± 14.20	+2.88%	0.34 (small)	[−0.82, 1.50]
Zhang	120°/s	Right	168.77 ± 12.60	172.30 ± 11.80	+2.09%	0.28 (small)	[−0.87, 1.43]
Zhang	150°/s	Left	174.50 ± 16.30	184.90 ± 15.10	+5.96%	0.64 (medium)	[−0.60, 1.88]
Zhang	150°/s	Right	168.93 ± 13.40	176.30 ± 12.50	+4.36%	0.56 (medium)	[−0.66, 1.78]
Zhang	180°/s	Left	172.83 ± 14.80	173.80 ± 13.90	+0.56%	0.11 (negligible)	[−1.03, 1.25]
Zhang	180°/s	Right	167.77 ± 13.20	175.13 ± 12.20	+4.38%	0.56 (medium)	[−0.66, 1.78]

**Table 4:** Trunk rotation peak power before and after the 15-week periodized strength training program.

Athlete	Velocity	Direction	Pre (M ± SD)	Post (M ± SD)	%Δ	Cohen’s d	95% CI for d
Fu	60°/s	Left	127.13 ± 8.90	148.27 ± 7.80	+16.63%	2.38 (large)	[0.16, 4.60]
Fu	60°/s	Right	79.73 ± 6.95	100.13 ± 6.20	+25.59%	2.93 (large)	[0.33, 5.53]
Fu	120°/s	Left	140.10 ± 23.80	156.60 ± 21.50	+11.78%	0.69 (medium)	[−0.57, 1.95]
Fu	120°/s	Right	89.30 ± 18.00	122.30 ± 16.20	+36.96%	1.83 (large)	[−0.02, 3.68]
Fu	150°/s	Left	164.07 ± 24.50	165.10 ± 23.10	+0.63%	0.05 (negligible)	[−1.08, 1.18]
Fu	150°/s	Right	118.23 ± 16.50	137.50 ± 15.20	+16.28%	1.16 (large)	[−0.30, 2.62]
Fu	180°/s	Left	158.17 ± 16.50	164.40 ± 15.80	+3.94%	0.38 (small)	[−0.79, 1.55]
Fu	180°/s	Right	114.40 ± 15.10	137.07 ± 14.20	+19.81%	1.50 (large)	[−0.15, 3.15]
Ren	60°/s	Left	98.53 ± 9.10	108.60 ± 8.50	+10.22%	1.11 (large)	[−0.33, 2.55]
Ren	60°/s	Right	81.07 ± 24.10	97.20 ± 20.50	+19.90%	0.67 (medium)	[−0.58, 1.92]
Ren	120°/s	Left	104.83 ± 26.30	123.67 ± 22.40	+17.97%	0.72 (medium)	[−0.55, 1.99]
Ren	120°/s	Right	78.27 ± 11.50	100.97 ± 9.80	+28.99%	1.97 (large)	[0.03, 3.91]
Ren	150°/s	Left	79.30 ± 9.40	105.17 ± 8.20	+32.62%	2.77 (large)	[0.28, 5.26]
Ren	150°/s	Right	73.60 ± 10.90	96.93 ± 9.40	+31.70%	2.13 (large)	[0.08, 4.18]
Ren	180°/s	Left	75.10 ± 9.40	100.17 ± 8.20	+33.38%	2.68 (large)	[0.26, 5.10]
Ren	180°/s	Right	75.57 ± 20.20	91.60 ± 17.50	+21.21%	0.80 (large)	[−0.50, 2.10]
Zhang	60°/s	Left	105.62 ± 15.10	125.81 ± 13.50	+19.12%	1.34 (large)	[−0.22, 2.90]
Zhang	60°/s	Right	74.32 ± 14.50	98.01 ± 13.20	+31.87%	1.63 (large)	[−0.10, 3.36]
Zhang	120°/s	Left	111.80 ± 19.50	137.50 ± 17.80	+22.99%	1.32 (large)	[−0.23, 2.87]
Zhang	120°/s	Right	87.40 ± 17.40	105.70 ± 15.80	+20.94%	1.06 (large)	[−0.35, 2.47]
Zhang	150°/s	Left	91.20 ± 18.60	125.93 ± 16.50	+38.08%	1.86 (large)	[−0.01, 3.73]
Zhang	150°/s	Right	79.50 ± 20.50	110.07 ± 18.30	+38.45%	1.49 (large)	[−0.15, 3.13]
Zhang	180°/s	Left	83.40 ± 19.30	106.50 ± 17.20	+27.70%	1.20 (large)	[−0.28, 2.68]
Zhang	180°/s	Right	78.60 ± 18.40	100.60 ± 16.50	+28.00%	1.20 (large)	[−0.28, 2.68]

**Table 5:** Left-right trunk rotation ratio before and after the 15-week periodized strength training program.

Athlete	Velocity	Pre (M ± SD)	Post (M ± SD)	%Δ	Cohen’s d	95% CI for d
Fu	60°/s	1.36 ± 0.10	1.38 ± 0.08	+1.47%	0.11 (negligible)	[−1.03, 1.25]
Fu	120°/s	1.39 ± 0.12	1.38 ± 0.10	−0.72%	0.00 (negligible)	[−1.13, 1.13]
Fu	150°/s	1.47 ± 0.13	1.37 ± 0.09	−6.80%	0.92 (large)	[−0.43, 2.27]
Fu	180°/s	1.36 ± 0.10	1.26 ± 0.08	−7.35%	1.00 (large)	[−0.39, 2.39]
Ren	60°/s	1.29 ± 0.13	1.34 ± 0.11	+3.88%	0.20 (small)	[−0.94, 1.34]
Ren	120°/s	1.27 ± 0.14	1.24 ± 0.10	−2.36%	0.43 (small)	[−0.75, 1.61]
Ren	150°/s	1.20 ± 0.12	1.22 ± 0.10	+1.67%	0.22 (small)	[−0.93, 1.37]
Ren	180°/s	1.19 ± 0.15	1.21 ± 0.10	+1.68%	0.07 (negligible)	[−1.06, 1.20]
Zhang	60°/s	1.12 ± 0.10	1.08 ± 0.08	−3.57%	0.40 (small)	[−0.78, 1.58]
Zhang	120°/s	1.07 ± 0.11	1.08 ± 0.09	+0.93%	0.10 (negligible)	[−1.03, 1.23]
Zhang	150°/s	1.03 ± 0.12	1.05 ± 0.10	+1.94%	0.09 (negligible)	[−1.04, 1.22]
Zhang	180°/s	1.03 ± 0.11	0.99 ± 0.10	−3.88%	0.50 (medium)	[−0.70, 1.70]

Note: (Applies to Table 3 and Table 4 and this table): Pre- and post-values are mean ± SD across the 3–5 maximal contractions retained for analysis. %Δ = percentage change from pre to post. Cohen’s d = (M_post − M_pre)/SD_pre, with magnitude: negligible |d| < 0.2, small 0.2–0.5, medium 0.5–0.8, large ≥ 0.8. 95% CI for d = d ± 1.96 × SE_d, where SE_d = sqrt(1/*n* + d^2^/(2*n*)) with *n* = 3; these intervals are unstable at *n* = 3 and should be interpreted with caution. No inferential tests were conducted.

## Data Availability

The raw data supporting the conclusions of this article will be made available by the corresponding author upon reasonable request, subject to ethical and privacy considerations.

## Supplementary Materials

The following supporting information can be downloaded at: https://www.mdpi.com/article/10.3390/jfmk11040389/s1, Table S1. Weekly Training Schedule; Table S2. Overall Training Plan.
