# Supplementary material for: Trunk Rotation Isokinetic Strength Changes During a 15-Week Periodized Strength Training Program in Three Chinese Elite Paralympic and Deaflympic Throwers
Source: J Funct Morphol Kinesiol. 2026 Sep 28;11(4):389. doi: 10.3390/jfmk11040389 (PMC13692408; doi:10.3390/jfmk11040389)
Supplement: Supplementary file 1 [file jfmk-11-00389-s001.zip › jfmk-4520882-supplementary.pdf]

Table S1. Weekly Training Schedule

**Day**	**Training Content**
Monday	Speed training
Tuesday	Sport-specific technique training
Wednesday	Strength training (upper limb, lower limb, and trunk strength)
Thursday	Ball games (to develop coordination and flexibility)
Friday	Sport-specific technique training
Saturday	Strength training
Sunday	Rest

Note: Athletes: Fu Xinhan, Wang Yanzhang, Huang Yuhua, Gu Yingqing, Ren Chunxia, Li Xia, Zuo Chuofeng, Xue Baowei, Li Zhihong, Liu Shaoqi, Cui Zhijie, Xiao Kangjia, Zhu Meichen. Coaches: Coach Zhang, Coach Shen, Coach Lin, Coach Gu. Intern: Su XuanYu.

Table S2. Overall Training Plan

Training Period	Training Sub-phase	Methods and Load
Preparation Period	Anatomical Adaptation Phase (2–4 weeks)	Engage most muscle groups; strengthen muscles, ligaments, and tendons. Core areas—including the abdominals, lower back, and spine—are also development priorities. **Training methods:** Upper body—snatch, clean and jerk, bench press, rapid presses, etc.; Core—crunches, leg raises, sit-ups with piked legs, etc.; Lower body—heel raises, squats, lunge walks, etc. **Load:** Throughout this phase, repetitions decrease from 12–20 down to 6–8. **Sets:** 6–8 reps × 2–4 sets. **Total training time:** 40 minutes. **Rest intervals between exercises:** 30–120 seconds. **Weekly frequency:** 3 or 4 times.
	Muscle Hypertrophy Phase (2–5 weeks)	6–9 types of specific or non-specific exercises for the primary muscle groups (specific exercises include: kettlebell exercises, band lateral push-offs; band lateral push-off and stand-up; single-arm barbell push (one end loaded); snatch; power clean; bench press, etc.). **Rest between sets:** 3–5 minutes. **Frequency:** 2–4 sessions/week. **Sets:** 4–6 sets/session. **Repetitions:** 10–15 reps/set. **Intensity:** 70%–80% of maximal strength.
	Maximal Strength Phase (3–5 weeks)	Exercises include: squats, bench press, lateral one-arm heavy kettlebell lifts, kettlebell/band standing presses, supine single-arm kettlebell pushes, etc. **Exercises:** 4–6 movements per session. **Frequency:** 2–3 sessions/week. **Sets:** 3–4 sets/session. **Intensity:** above 80% of maximal strength.
Competition Period	Explosive Power Conversion Phase (1–2 weeks	This phase is not a sudden switch but rather a gradual transition from maximal strength to explosive power. **Training means:** Specific or sport-specific movements (e.g., for shot put: sideways kettlebell push; band pull and

Training Period	Training Sub-phase	Methods and Load
	transition; 1–2 weeks conversion)	lateral push-off; standing kettlebell push, etc.). **Load:** Moderate weight, with movements performed explosively and at speed. **Rest between sets:** 2–4 minutes. A certain proportion of maximal strength training should still be maintained. **Frequency:** 1–2 sessions/week. **Sets:** 3–4 sets/session. **Repetitions:** 8–15 reps/set. **Intensity:** 70%–80% of maximal strength. **Rest between sets:** 3 minutes. This training stage aims to preserve the athletes' competition energy and bring their sport-specific biomotor abilities to peak levels.
Transition Period	Recovery Phase	Training primarily consists of low-load exercises, sport-specific strength work, and relaxation/stretching methods. **Frequency:** 1–2 maximal strength sessions and 1–2 explosive power sessions per week. **Sets:** 1–2 sets/session. **Intensity:** approximately 65% of maximal strength. **Rest between sets:** 3–5 minutes. The objective is to eliminate fatigue accumulated during the training period by reducing training volume (through decreased frequency) and lowering training intensity, while replenishing depleted energy stores.
